# Observations on the Occurrence, Transmission and Management of the COVID-19 Pandemic Derived from Physics

**DOI:** 10.3390/diseases9010009

**Published:** 2021-01-16

**Authors:** John G. Ingersoll

**Affiliations:** ECOCORP, 1211 South Eads Street, Suite 803, Arlington, VA 22202, USA; jgingersoll@ecocorp.com; Tel.: +1-202-999-0840

**Keywords:** COVID-19, SARS-CoV-2, nature, symbiosis, quantum world, superspreader, infection pathway, ACE-2 marker, power law, morbidity risk index

## Abstract

Three important observations derived from the ongoing COVID-19 pandemic could result in the development of novel approaches to deal with it and avoid or at least minimize the occurrence and impact of future outbreaks. First, the dramatic increase in pandemics in the past decade alone suggests that the current relationship of humans with the environment is quickly becoming unstable, with potentially catastrophic consequences. In order to reduce the toll in life and property, we would need to shift our emphasis from control of nature to a symbiosis with nature. This, then, can become the new framework for dealing effectively with environmental issues such as climate change, whereby properly applied medical science would provide the necessary impetus for action. Second, the existence of superspreaders of infection among populations in this pandemic requires that we develop objective tests, most likely of a genetic nature, to identify them rather than apply indiscriminate and draconian controls across the board. Not identifying superspreaders in a timely fashion could allow this pandemic to turn into a black swan event, with a catastrophic impact on society. Third, we need to refocus our efforts in dealing with this pandemic from the virus itself to the human hosts. An objective morbidity risk index can be developed such that most of us can go about our daily business without the fear of becoming seriously ill, while measures can be implemented to protect those who are most vulnerable to this virus. These observations point clearly to a need for a paradigm shift.

## 1. Introduction

Infectious diseases have been around at least since the beginning of the Neolithic era some 12,000 years ago, when humans started to settle into communities, domesticate animals and grow crops [1]. The COVID-19 pandemic demonstrates the power of a tiny organism, in this case the virus SARS-CoV-2, to disrupt the lives of billions of humans across the globe and create economic losses amounting to tens of trillions of dollars and still rising. The response across the board among scientists, health professionals and the general public has been almost unanimous and entirely predictable: find ways to control the virus, get rid of it altogether and return to a business-as-usual existence as soon as possible. It is not uncommon for elected officials and ordinary citizens alike to speak of the war against the virus; while the current war-like approach to dealing with this virus may or may not succeed, it potentially reveals a deep misunderstanding of the presence of the virus among us.

As is common in human reasoning, cause and effect with regards to pandemics tend to be reversed. In reality, the virus has arrived among us because we humans have unwittingly extended an invitation to it. Enlightened members of the scientific community, and undoubtedly a large number of the public, already recognize this aspect of the appearance of the virus [2]. The sooner we realize this fact across the board, the better we will be equipped to deal with the pandemic situation most effectively at every level, from the individual to the societal. This, of course, implies that we must refocus our efforts away from the virus and onto us.

## 2. Observations

According to the WHO, infectious diseases are responsible for 20% of deaths across the globe, amounting to over 11 million annually [3]. The COVID-19 pandemic will increase these figures [4], but it is still nowhere close to the 1918 influenza pandemic that killed an estimated 50 million [2] or to the earlier recorded pandemics of 541 AD (Justinian Plague) and of 1348 AD (Black Death), each of which killed an estimated 1/3rd of the populations in Eurasia [5,6]. In the case of Black Death, 75 to 150 million people may have perished. The SARS-CoV-2 virus has the potential to create another major pandemic, because it has not infected humans before in a sustained manner, it has an extremely high degree of transmission and it has a relatively high rate of mortality [2]. Moreover, if it is found that either the virus never leaves an infected person, but rather remains dormant in the body, or if the immunity to infection is very short-lived, then the COVID-19 pandemic will be around for a long time.

The first observation has to do with the appreciation that all living organisms, along with all inanimate objects, i.e., all of the natural world, exist in an interdependent and interconnected state, or in a symbiotic manner to use a term from biology. Interconnectedness is the key tenet of our quantum world that has been gradually replacing, over the past 100 years, the earlier concept of a mechanistic world [7,8]. We may also note that the notion held widely among biologists that quantum physics is not applicable in their field has been demonstrated to be incorrect [9]. The idea of interconnectedness in nature was observed, studied and published extensively by Alexander von Humboldt as early as the first half of the 19th century, i.e., well before quantum physics, although it was later put aside, most likely for political reasons [10,11,12]. Humans can control at will a mechanistic world, but they have to live in harmony within the quantum world, within a cosmos in which they are an integral component. Moreover, while the mechanistic or reductionist approach has had a tremendous impact on the well-being of humans, particularly in regards to the advances in medicine, it may now be reaching its useful limit.

The limit to the reductionist approach is becoming apparent from the explosive increase in potentially pandemic infectious diseases in the 21st century such as SARS (2002), H1N1 (2009), MERS (2012), Chikungunya (2014), Zika (2015), the recurrence of Ebola (2014) and now COVID-19 [2]. All these diseases, as well as the aforementioned earlier pandemics, either originated in animals or have employed animals for their transmission. Bats and pangolins are believed to be the animal culprits in COVID-19 [13]. The link of human infection to an animal pathogen was expounded scientifically in the middle of the 19th century by Rudolph Virchow, who coined the term “zoonosis” to describe it [14]. Yet, more than 150 years later, we remain by-and-large oblivious to it, as manifested by our human-dominated interaction with the environment. It is reported that the Black Death, in its initial phase, lasted for about five years, after which it kept coming back periodically, albeit not as virulent, for the next 400 years [6]. Depending on how long the COVID-19 pandemic persists and whether an effective vaccine against it can be developed, humans may have to seriously rethink their relationship with nature sooner rather than later.

This new relationship may entail among others the following: people moving away from dense metropolitan centers and back into the country; the reduction in commuting for work and for shopping if such activities can be carried out remotely from home; a revision of social interactions, whereby families can cluster together; increased efficiency in the use of energy, water and food; more effective recycling and reuse of wastes; local production of food to the extent possible. The debate on climate change over the past several decades has had a limited degree of acceptance among the public, perhaps because it is framed in the wrong way [15]. The choice of framework is crucial not only for communicating the impact, but also for implementing a solution. The term “climate change” over the past several decades conveys little about its impact and even less about its solution. Change is unavoidable and occurs naturally all around us. Moreover, an associated remedy brought up by the term carbon tax sounds ominous as indicative of the state raising taxes that are perceived as going to be wasted. The alternative framing of the problem of global warming emphasizes only one aspect of the impact that is not readily witnessed from year to year due to weather variability, and creates a negative, i.e., undesirable, feeling among the public.

In the middle of the 19th century, Rudolf Virchow, a physician, anthropologist, biologist, historian, writer and politician, who is known as the father of modern pathology and the founder of social medicine, coined the well-known aphorism: “Medicine is a social science, and politics is nothing else but medicine on a large scale” [16,17]. Even though in recent times the emphasis of medicine has been on treating disease, the revolutionary accomplishment of medicine since the mid-19th century has been the avoidance of disease. The current pandemic can reorient the emphasis of medicine to bring it back to prevention, but at a grand scale, encompassing the interconnectedness of humans with themselves and the rest of nature. Thus, social, holistic or quantum medicine can become the effective, i.e., persuasive, means of action for the public at large to recognize, accept and live in the new framework of “symbiosis with nature.”

**Figure 1:**
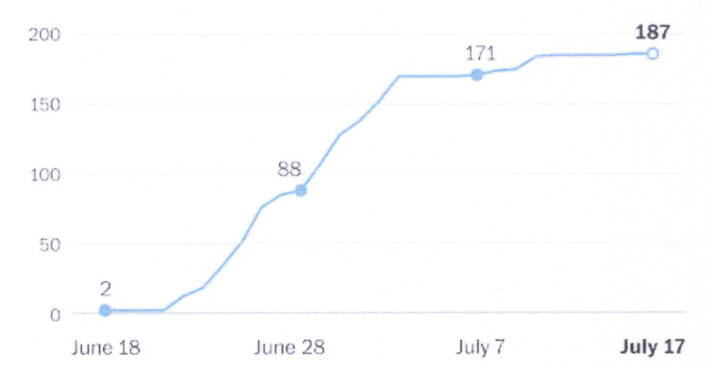
Example of COVID-19 cluster outbreak in Lansing, Michigan at a restaurant and pub by a few infected superspreaders (Source: Ingham County Health Department).

The second observation from the COVID-19 pandemic is the realization of the existence of superspreaders of the infection among the population [18,19]. That is to say, while most infected people may infect only a small number of people, a few infected persons can infect a very large number of people, creating what is called a ‘cluster event’. Cluster events have occurred aboard ships, at nursing homes, meatpacking plants, ski resorts, churches, restaurants, hospitals and prisons and are manifested by the nature of the occurrence of infection among the general population. An example is shown in Figure 1. Epidemiologists use the reproduction number R to describe the average number of new infections caused by each infected person. For most people, the R will be zero, but for the superspreaders, it will be quite high. For SARS-CoV-2, the average value of R without social distancing is estimated to be between 2 and 3, at least in the early stages of the pandemic, although the R value of the recently discovered fast-spreading variant is estimated to be 0.4 higher [20]. By way of comparison, the R value for the 1918 influenza pandemic has been estimated to be 1.80, with an interquartile range from 1.47 to 2.27 [21]. In addition to the R value, a parameter called the dispersion factor k is used to describe how much a disease clusters. The lower the k is, the more transmission is caused by a small number of people. For example, the estimated value of k for SARS was 0.16, for MERS 0.25 and for the 1918 influenza pandemic close to 1. For SARS-CoV-2, the current estimates of k vary from 0.10 to 0.44 [18,22]. This suggests that superspreaders are not important for influenza, but they are highly important for SARS-CoV-2. Moreover, if it is conclusively established that most transmissions occur during the pre-symptomatic phase of infection, then SARS-CoV-2 becomes difficult to contain, as computer simulations have shown [23]. In that instance, the COVID-19 pandemic may be evolving into the perfect storm [2].

**Figure 2:**
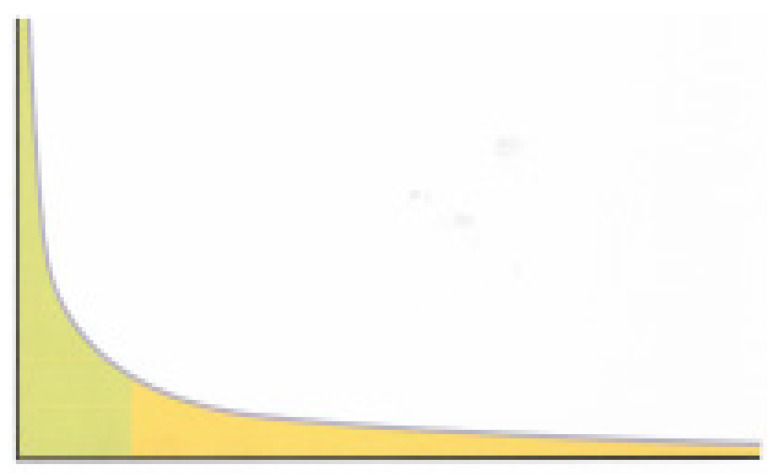
Power law: horizontal axis—persons; vertical axis—number of infections attributed to a particular person; green area—infections by superspreaders—roughly 20% of persons account for about 80% of total infections; yellow area—infections by all other persons—roughly 80% of persons account for 20% of total infections.

The occurrence of superspreaders in the COVID-19 pandemic is indicative of a power law statistical distribution for the occurrence of the disease. In a power law distribution, shown schematically in Figure 2, the functional relationship between two quantities is such that a change in one quantity results in a proportional relative change to the other quantity, independent of the initial size of those quantities. Power laws are ubiquitous in nature (some examples are given in parentheses) whether it be in physics (black body radiation, quantum mechanics), economics (income and wealth distributions, cost of health benefits per person), finance (business income per client, contribution of taxes per person), geography (size of cities), geology (size of lakes and mountains, volcanic eruptions), environmental quality (emissions by cars and power plants), linguistics (occurrence of words and letters), sociology (criminal charges per convict), ecology (number of distinct species per ecosystem) and so on.

A power law is also known as a Pareto law, because Vilfredo Pareto was first to articulate it at the turn of the 20th century while describing wealth distribution. The Pareto law is also known as the law of 80/20, because typically, 20% of the input is responsible for 80% of the outcome. In the case of the COVID-19 pandemic, indications are that between 10 and 20% of those infected generate 80% of subsequent infections [18,19]. A very important characteristic of a power law is that it is scale invariant. This means that depending on the parameters of the power law, it may or may not have a well-defined mean and a finite variance. As it turns out, most power laws occurring in nature have a well-defined mean but not a finite variance. A lack of a finite variance can lead to a so-called black swan event, an event that is rare and unexpected, has a huge impact and can be explained in hindsight [24,25]. The values of R and k for COVID-19 will determine whether the current pandemic can lead into a black swan event, but since we may not know that for some time, if ever, it is important to develop new measures to reduce the likelihood of such an outcome.

Clearly, the present “3 C’s” approach to suppress the impact of superspreaders by imposing on everybody the avoidance of (a) closed spaces with poor ventilation, (b) crowded settings and (c) close contact with others is not a long-term solution [19]. Contact tracing, even though useful in curbing the spread of the virus, has its limitations in tracking asymptomatic persons, including superspreaders. This has been demonstrated clearly in Germany, where contact tracing is credited for relatively lower infection and death rates, but still failed to identify in 65% of the cases how a person got infected [26]. The new measures to be developed would include the identification via objective means, i.e., testing of most likely genetic basis, of the superspreaders such that their contribution to additional infections can be effectively controlled [27]. It is already established that the gateway for invasion by SARS-CoV-2 into human host cells is via the angiotensin-converting enzyme 2 (ACE-2) receptor [28]. Consequently, SARS-CoV-2 has the unusual capacity to attack many different types of human host cells and tissues simultaneously in the respiratory, cardiovascular, gastro-intestinal, renal-excretory, reproductive and central nervous systems, thereby becoming injurious to diverse cells, tissues and organ systems and exploiting any immune weakness in the host [29]. The variability in the expression of the ACE-2 receptors among individuals, and hence, the potential for SARS-CoV-2 infection and severity as manifested by age, sex, ethnicity, and several co-morbidities, such as cardiovascular disease, cancer, metabolic syndrome and obesity, and cognitive decline, may also be critical in the genetic constitution, and hence, identification of the superspreaders. Thus, there are may be additional, e.g., GI-tract, and more effective, i.e., higher viral load, pathways for the transmission of infection than the presently emphasized respiratory path through the so-called Flügge’s droplets, produced from breathing, talking, sneezing and coughing [29]. The mapping of the expression of the ACE-2 receptors in superspreaders may form the basis of the genetic identification of these individuals in the population.

We may also note that the reason power laws are so prevalent is that nature confers equal chance of outcome to every actor, animate or inanimate, within a particular situation. In a pandemic, everybody is given the same chance of infecting someone else, and as a result, we obtain the most probable distribution of the majority of people not contributing to the transmission and only a relatively small percentage affecting it. Even though this reason seems counterintuitive, it is borne out of experience in quantum physics whereby the derivation of Planck’s black body radiation law is based on the assumption of the equal access to energy among all oscillators (actors) maximizing (reflecting the most probable outcome) the system entropy (indicative of state of equilibrium) [8,30]. Thus, symbiosis with nature is at work here as well.

The third observation from the COVID-19 pandemic is that thus far, we have focused too much on the virus and not enough on the person getting infected: the “host”. It has already been established that certain characteristics as well as pre-existing conditions of human hosts make them more susceptible to severe illness and increased risk of death. These include: age (85 or older), because of the normally occurring immune system senescence; obesity (body mass index above 30 kg/m^2^); blood type (type O offers increased protection); cardio-vascular disease (heart failure, coronary artery disease, cardiomyopathies, pulmonary hypertension); cancer; diabetes (type 2); chronic kidney disease; chronic obstructive pulmonary disease; immune compromised state; sickle cell disease; pregnancy; gender (male or female); race; and perhaps other conditions to be determined as we acquire a better understanding of the virus [2,31,32].

Thus, the SARS-CoV-2 virus can cause severe disease and even death to those who happen to be afflicted by any of the aforementioned conditions or co-morbidities at any age, although the occurrence of such co-morbidities increases naturally with age as the body fails along with the immune system losing some of its potency, i.e., developing senescence. It is interesting to note that the COVID-19 pandemic clearly reconfirms both aspects of the 19th century debate on the causality of disease: Louis Pasteur and Robert Koch demonstrated that a microbe was the necessary agent, while Rudolf Virchow insisted that only those with compromised cells would become ill [14]. Naturally, both sides were correct in understanding the nature of an infectious disease. No one would disagree that each host is unique in so far as how their immune system and body would respond to the virus, a clear indication of a quantum effect. However, the complexity of the immune system, which may never be fully understood at the microscopic level, should not stop us from taking action, as there are still general macroscopic markers that can be employed to assign a degree of morbidity risk with respect to the virus to each individual. These markers comprise the aforementioned macroscopic conditions that SARS-CoV-2 exploits. Based on a statistical analysis of the data on the millions of infected people and the hundreds of thousands of them who became severely ill and even succumbed to the disease, even if the data are not perfect, one can develop a quasi-quantitative numerical risk index, say, a number between 0 to 100, of a person’s degree of morbidity risk. Those with a higher risk index would need to be shielded against exposure, while those with a lower risk index could resume normal activities without the fear of getting seriously ill. Such a system could be readily implemented via the internet and through social media at a much faster pace, at a much lower cost and most likely with higher efficacy than, for example, a vaccine. The proposed re-focusing on the human host will serve well to complement the administration of a vaccine, even if the latter is highly efficacious. This approach will also enable us to obtain herd immunity while protecting those who are most vulnerable. The age-old concept of herd immunity refers to the protection conferred by immune individuals to the susceptible ones in a given population by limiting the spread of the disease. The percentage of the population Y required to be immune through infection or vaccination is a function of the reproduction number R and is calculated as follows: Y = (R − 1)/R × 100 [33]. For SARS-CoV-2, the required herd immunity may be as low as 50% for R = 2 to as high as 70.5% for R = 3.4, reflecting the new variant of the virus. We may also note that even in the deadliest pandemics, certain members of the population manage to not be infected, even though they are in the midst of infected people. This observation has been reaffirmed in the current pandemic, where among people in close proximity, such as the members of a family, some have been infected and even succumbed and others do not get infected [34]. This would seem to defy the traditional cause and effect mechanistic view of infection. The question, then, is whether there is another, as yet unknown, but profound, quantum in nature, for example, a psychosomatic connection between the human host and the infectious agent that can be ascertained [8,35,36]. Perhaps, the interaction between the host and the virus occurs not just at the reductionist level of cause and effect, but rather at a quantum level described by the quantum concepts of interconnectedness and entanglement.

## 3. Conclusions

In this study, we have briefly addressed three observations derived from the current pandemic and have presented an alternative course of action applicable not only to the current situation but also to future occurrences. First, we would need to reconsider our relationship with nature from that of control to one of symbiosis. Depending on how the current pandemic evolves, we may be forced into symbiosis sooner rather than later. After all, the popular adage “our actions will destroy nature” is false. Rather, we should realize that it is “if we do not heed the warnings of nature, we will end up being destroyed”. Second, regarding the existence of superspreaders in the current pandemic, we would need to expand our understanding of transmission pathways and develop suitable objective genetic tests, to identify those among the human hosts that are the superspreaders. Third, we should focus our efforts much more on the human hosts, i.e., us, instead of the agent, i.e., the virus, in order to develop a prescriptive morbidity risk index for every human host. Discriminating policies would then be implemented to protect those at higher risk, while those at lower risk can go about their lives without fear and anti-social restrictions. Such policies ought to and will complement rather than compete with other approaches such as the development and administration of vaccines. The three points are seemingly unrelated, but are in fact highly interrelated observations from a quantum physics perspective, and point to the need for a paradigm shift in our interaction with nature. The current approach of command and control will have to be superseded by a new approach that can be framed as a symbiosis with nature, with medical science properly implemented for its realization.

## Data Availability

Not applicable.
